# A novel gas ionization sensor using Pd nanoparticle-capped ZnO

**DOI:** 10.1186/1556-276X-6-534

**Published:** 2011-09-30

**Authors:** Hongjun Wang, Changwei Zou, Canxin Tian, Lin Zhou, Zesong Wang, Dejun Fu

**Affiliations:** 1 Department of Physics, Wuhan University, Wuhan 430072, China; 2 Department of Physics, Zhanjiang Normal University, Zhanjiang 524037, China; 3 Key Laboratory of Beam Technology and Materials Modification of Ministry of Education, Beijing Normal University, Beijing 100875, China

## Abstract

A novel gas ionization sensor using Pd nanoparticle-capped ZnO (Pd/ZnO) nanorods as the anode is proposed. The Pd/ZnO nanorod-based sensors, compared with the bare ZnO nanorod, show lower breakdown voltage for the detected gases with good sensitivity and selectivity. Moreover, the sensors exhibit stable performance after more than 200 tests for both inert and active gases. The simple, low-cost, Pd/ZnO nanorod-based field-ionization gas sensors presented in this study have potential applications in the field of gas sensor devices.

## 1. Introduction

Gas sensors have attracted considerable attention in recent years because of their huge potential applications, such as pollution detection, environment protection, gas detection for counter-terrorism, etc. [1]. There are two types of gas sensors, chemical type operated by gas adsorption-desorption and physical type operated by field ionization. In different gas varies and concentrations atmosphere, the chemical type gas sensor can detect the modifications of the electronic properties in the active layer, such as carbon nanotubes (CNTs) [2-4], porous silicon [5], and metal oxides [6]. However, the application of the chemical type gas sensor is limited by several disadvantages, such as the potential difficulties in detecting gases with low adsorption energies, the high working temperature (except for the CNTs based sensors), and the higher power consumption.

Recent efforts have been directed to the physical type of gas senor based on the field ionization, which works by fingerprinting the ionization characteristics of distinct gases. This type sensor can detect gases regardless of their adsorption energies. A novel physical gas sensor based on CNTs has been demonstrated with low breakdown voltage due to its extremely sharp radii [7,8]. This sensor can detect many gases, such as Air, He, Ar, and gas mixtures, by the strong electric fields generated at the tips to strip electrons from the various gas molecules [7]. However, the CNTs show poor stability because it could easily be oxidized and degraded in the oxygen-contained atmosphere [9,10].

Recently, gas ionization sensors using a sparse array of vertically aligned gold nanorods as substitutes for CNTs have successfully been prepared for the first time [11-14]. Owing to the chemical stability of one-dimensional ZnO (1D ZnO) nanowires at room temperature, they also have been used for stable field-ionization gas sensors instead of CNTs [10]. However, 1D ZnO nanostructures with relatively smooth surface and larger tip radii, compared with CNTs, need higher breakdown voltage. Therefore, the modification of the surface of 1D ZnO nanostructures to obtain lower breakdown voltage is one of the key issues for gas sensor applications. In this study, we introduce a physical gas sensor using palladium (Pd) nanoparticle-capped ZnO (Pd/ZnO) nanorods as the anode. The results show that the breakdown voltage decreases for the Pd/ZnO nanorod-based sensor, compared with the bare ZnO nanorod. This study investigates the potential applications of such physical ionization gas sensors.

## 2. Experimental section

ZnO nanorods were grown on silicon substrates through a reactive vapor deposition method as reported in detail elsewhere [15]. The Pd nanoparticles were deposited by a dc sputtering system at a fixed current of 40 mA for 120 s. The morphology of ZnO nanorods was characterized by a Sirion FEG scanning electron microscopy (SEM) and JEM-2010FET transmission electron microscope system operated at 200 kV, respectively. All the data were obtained from the same ZnO nanorods sample, which was cut into two pieces before Pd sputtering.

**Figure 1:**
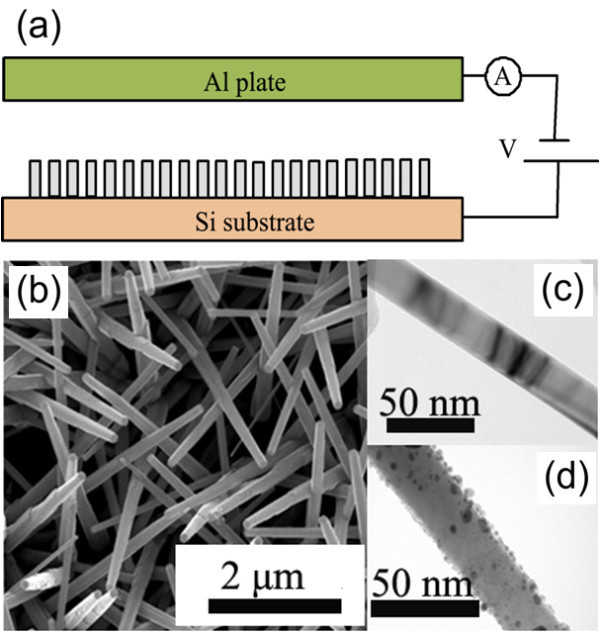
**(a) Schematic diagram of the ZnO nanorod-based gas sensor device**. **(b) **SEM image of ZnO nanorods. TEM images of the nanorods: **(c) **Individual bare ZnO nanorod and **(d) **Pd/ZnO nanorod with Pd capping by 120 s sputtering, respectively.

Schematic illustration of the ZnO nanorod-based gas sensor device is shown in Figure 1a. It consists of two electrodes, anode (ZnO nanorods) and cathode (Al plate). An insulated plastic thin film was used to adjust the distance between the two electrodes. In this experiment, the space distance is set to 500 μm and the effective area is 0.5 × 0.5 cm^2^. The voltage of the two electrodes can be varied from 0 V to 20 kV (DW-P203), and the current is measured by a multimeter (Mastech MS8040). Prior to experiment, the base pressure of the chamber was pumped to 1 × 10^-4 ^Pa.

## 3. Results and discussion

Figure 1b shows the top view SEM image of the bare ZnO nanorods. The ZnO nanorods have a diameter of 30-40 nm and length of 1 μm. Figure 1c, d shows the typical individual bare ZnO nanorods and Pd/ZnO nanorods with Pd capping by 120 s sputtering, respectively. It is clearly seen that the bare ZnO nanorod has a rather smooth surface and the surface of Pd/ZnO nanorod is distributed with Pd nanoparticles with diameters of about 5 nm.

**Figure 2:**
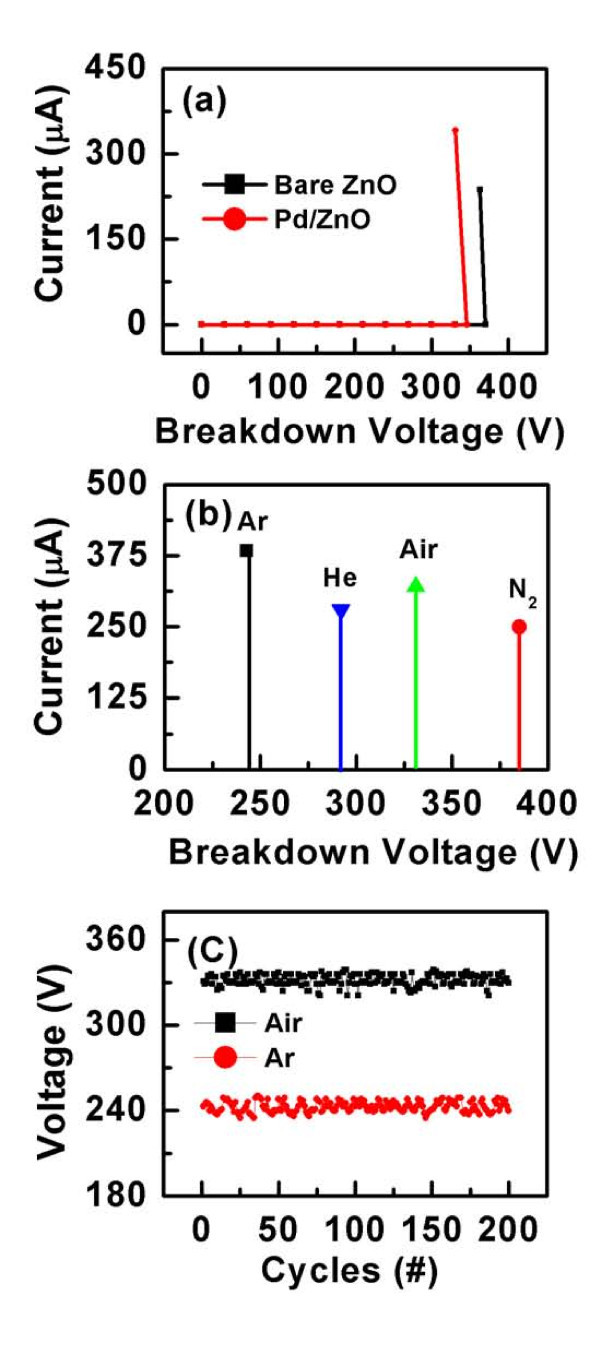
**Current-voltage (I-V) curves of the senors**. **(a) ***I*-*V *curves of the gas sensors using bare ZnO and Pd/ZnO nanorods and **(b) ***I*-*V *curves of Pd/ZnO nanorod-based sensors for Ar, He/CH_4_, Air, and N_2_, showing distinct breakdown voltage. **(c) **The stability tests of Pd/ZnO nanorod-based sensors for air and Ar.

The device was first tested in air under 100 Pa with anode-cathode separation of 500 μm using bare ZnO and Pd/ZnO nanorods (Figure 2a). A continuous current discharge of 273 μA was generated for the bare ZnO nanorods at 363 V, whereas a higher current of 341 μA was observed at a lower breakdown voltage of 341 V using Pd/ZnO nanorods. The same test was also carried out by replacing ZnO nanorods with Al plate and the breakdown voltage occurred at 1532 V with a current discharge of 65 μA (data not shown here). The results show that by the use of Pd/ZnO nanorods as anode, compared with bare ZnO nanorod, the breakdown voltage of air was reduced. Besides that, the discharge current was also increased, indicating the high sensitivity of sensors using ZnO nanorods. This is because the Pd nanoparticles act as the role of protuberances on the smooth surface of nanorods, which can create higher nonlinear electric field at the top nanoparticles than other smooth surface. This speeds the occurrence of breakdown process [1].

Figure 2b shows the breakdown voltage of the sensor using Pd/ZnO nanorods for Ar, He/CH_4 _(60%/40%), Air, and N_2_, respectively. All the tests were performed at room temperature and at a chamber pressure of 100 Pa. It can be seen that each gas exhibits a distinct breakdown voltage: Ar shows the lowest breakdown voltage and N_2 _displays the highest one. This precise breakdown voltage is a fingerprinting property for individual gas. The stability of sensor using Pd/ZnO nanorods was tested for air and insert gas of Ar, as shown in Figure 2c. The breakdown voltages of both gases are maintained up to 200 cycles without any significant change. It shows that the sensor exhibits much better stability than that of CNTs [10]. The good performance indicates that Pd/ZnO nanorods could be a better candidate for the field-ionization gas sensor.

**Figure 3:**
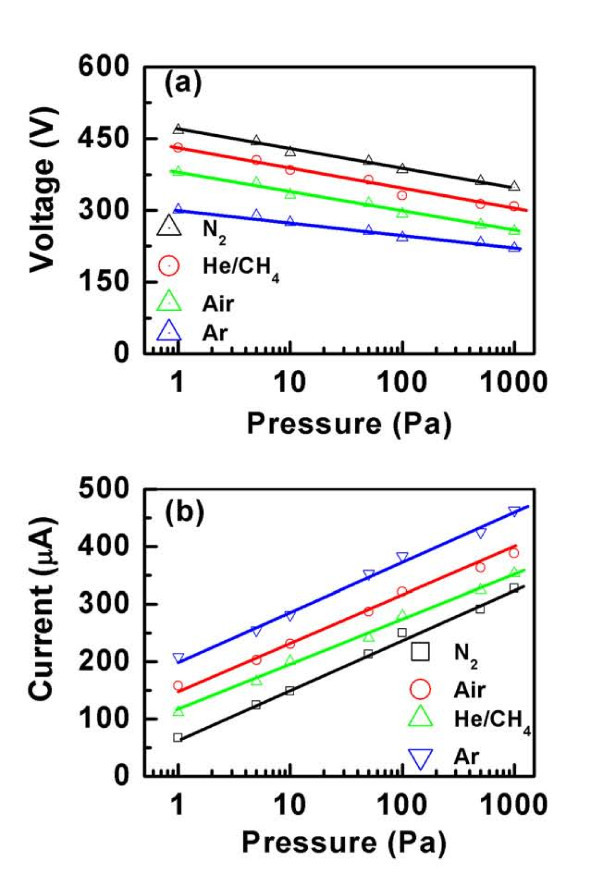
**Effect of gas pressure on electrical breakdown for the Pd/ZnO nanorod based sensor**. **(a) **Breakdown voltage versus gas pressure. **(b) **Discharge current at breakdown voltage versus gas pressure.

To study the effect of pressure on the electrical breakdown behavior of Pd/ZnO nanorod-based sensor, tests were performed at different pressures (Figure 3). The effect of pressure on the breakdown voltage of Air, N_2_, He/CH_4 _(60%/40%), and Ar is shown in Figure 3a. Note that the breakdown voltage of all the gases increases with decreasing pressure. This is because that the mean-free path of the electron is reduced at higher pressure. As a result, the higher energies are required for the electrons to make inelastic collisions which can lead to breakdown. However, the breakdown voltage increases slightly with increasing pressure. This is because that the electrical breakdown behavior is dominated by the nonlinear electric field. The same observation is also discussed for the sensor using ZnO nanowires [15]. Figure 3b shows the discharge current at breakdown voltage as a function of pressure. Note that the discharge current varies nearly logarithmically with pressure over a wide range from 1 to 1000 Pa. This indicates that the discharge current at certain breakdown voltage is a characteristic property of the gas molecule density that contributes to the conduction. Therefore, the discharge current property provides a convenient way to quantify the gas pressure of the species that being detected.

**Figure 4:**
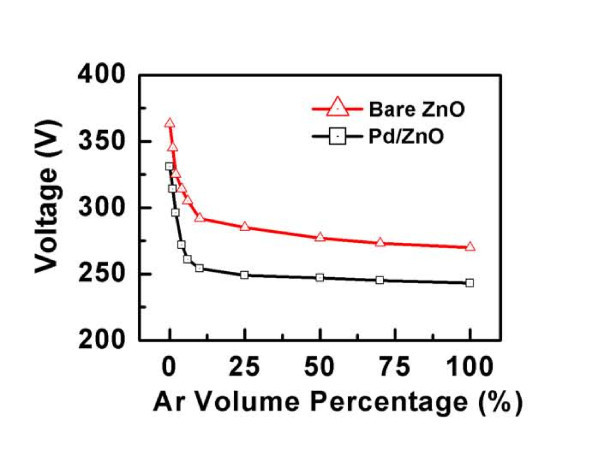
**Breakdown voltage of Ar in a mixture with air as a function of volume percentage under a constant 100 Pa pressure for the bare ZnO nanorod and Pd/ZnO nanorod-based sensors, respectively**.

To study the ability of monitoring gas mixtures without the direct use of chromatography arrangement, the mixture of Ar in air was tested using ZnO nanorod-based sensors. Figure 4 shows the breakdown voltage as a function of the relative percentage of Ar in the Ar-air mixture under a constant 100 Pa pressure for both bare ZnO and Pd/ZnO nanorod-based sensors. For the mixture containing over 50% Ar, the breakdown voltage nearly equals that of pure He for both bare ZnO and Pd/ZnO nanorod-based sensors. As the relative percentage of Ar in air reduced, the breakdown voltage increases from 277 (for 50% Ar) to 325 V (for 2% Ar) for the bare ZnO. However, a smaller breakdown voltage increase from 247 to 296 V is observed for the Pd/ZnO nanorods. This is because of the higher breakdown voltage of Air than Ar, which tends to impede the breakdown of Ar molecules. For both sensors below 2% of Ar in the mixture, the breakdown of Ar ceases and the breakdown voltage sharply rises. Similar results were also obtained for N_2 _and He/CH_4 _in a mixture with air. It indicates that Pd/ZnO nanorods, compared with the bare ZnO nanorods, show the same effective ability to quantify the concentration of different component in the mixture with a smaller breakdown voltage.

## 4. Conclusion

In conclusion, a novel field-ionization gas sensor using ZnO nanorods was demonstrated. The sensors using Pd nanoparticle-capped ZnO nanorods, compared with the bare ZnO nanorods, showed lower breakdown voltage. Besides that, the sensors showed good sensitivity and selectivity. Moreover, the breakdown voltage of Pd/ZnO nanorod-based sensors was maintained without any significant change during 200 cycle tests. The simple, low-cost devices presented in this study might be expected to expand the applications of gas sensor.

## Abbreviations

1D ZnO: one-dimensional ZnO; CNTs: carbon nanotubes; Pd: palladium; Pd/ZnO: Pd nanoparticle-capped ZnO; SEM: scanning electron microscopy.

## Competing interests

The authors declare that they have no competing interests.

## Authors' contributions

HW designed the experiments, carried out the sample preparation, performed the measurements, and drafted the manuscript. DF coordinated the research fund and activity and helped design the experiments. Both authors took part in the discussion of the results and helped shape the final manuscript. All authors read and approved the final manuscript.

## References

[B1] Hui GH Wu LL Pan M Chen YQ Li T Zhang XB A novel gas-ionization sensor based on aligned multi-walled carbon nanotubes Meas Sci Technol 2006 17 2799 10.1088/0957-0233/17/10/034

[B2] Collins P Bradley K Ishigami M Zettl A Extreme oxygen sensitivity of electronic properties of carbon nanotubes Science 2000 287 1801 10.1126/science.287.5459.1801 10710305

[B3] Sinha N Ma Z Yeow T Carbon nanotube-based sensors J Nanosci Nanotechnol 2006 6 573 10.1166/jnn.2006.121 16573108

[B4] Ong K Zeng K Grimes C A wireless, passive carbon nanotube-based gas sensor IEEE Sensor J 2002 2 82 10.1109/JSEN.2002.1000247

[B5] Kim SJ Lee SH Lee CJ Organic vapour sensing by current response of porous silicon layer J Phys D Appl Phys 2001 34 3505 10.1088/0022-3727/34/24/314

[B6] Zhang Y Xu JQ Xiang Q Li H Pan QY Xu PC Brush-like hierarchical ZnO nanostructures: synthesis, photoluminescence and gas sensor properties J Phys Chem C 2009 113 3430 10.1021/jp8092258

[B7] Modi A Koratkar N Lass E Wei BQ Ajayan PM Miniaturized gas ionization sensors using carbon nanotubes Nature 2003 424 171 10.1038/nature01777 12853951

[B8] Riley DJ Mann M MacLaren DA Dastoor PC Allison W Teo KBK Amaratunga GAJ Milne W Helium detection via field ionization from carbon nanotubes Nano Lett 2003 3 1455 10.1021/nl034460c

[B9] Wang MS Peng LM Wang JY Chen Q Electron field emission characteristics and field evaporation of a single carbon nanotube J Phys Chem B 2005 109 110 10.1021/jp046526d 16850991

[B10] Liao L Lu HB Shuai M Li JC Liu YL Liu C Shen ZX Yu T A novel gas sensor based on field ionization from ZnO nanowires: moderate working voltage and high stability Nanotechnology 2008 19 175501 10.1088/0957-4484/19/17/175501 21825672

[B11] Banan-Sadeghian R Kahrizi M A novel miniature gas ionization sensor based on freestanding gold nanowires Sens Actuators A 2007 137 248 10.1016/j.sna.2007.03.010

[B12] Banan-Sadeghian R Kahrizi M A novel gas sensor based on tunneling-field-ionization on whisker-covered gold nanowires IEEE Sens J 2007 8 161

[B13] Banan-Sadeghian R Kahrizi M A low pressure gas ionization sensor using freestanding gold nanowires IEEE ISIE 2007 1387 1390

[B14] Banan-Sadeghian R Kahrizi M A low voltage gas ionization sensor based on sparse gold nanorods IEEE Sens Conf 2007 648 651

[B15] Liao L Liu HD Li JC Liu C Fu Q Ye MS Synthesis and Raman analysis of 1D-ZnO nanostructure via vapor phase growth Appl Surf Sci 2005 240 175 10.1016/j.apsusc.2004.06.053

